# Green Synthesis of Silver Nanoparticles Using *Salvia verticillata* and *Filipendula ulmaria* Extracts: Optimization of Synthesis, Biological Activities, and Catalytic Properties

**DOI:** 10.3390/molecules28020808

**Published:** 2023-01-13

**Authors:** Vladimir Mihailović, Nikola Srećković, Zoran P. Nedić, Silvana Dimitrijević, Miloš Matić, Ana Obradović, Dragica Selaković, Gvozden Rosić, Jelena S. Katanić Stanković

**Affiliations:** 1 Department of Chemistry, Faculty of Science, University of Kragujevac, 34000 Kragujevac, Serbia; 2 Faculty of Physical Chemistry, University of Belgrade, 11159 Belgrade, Serbia; 3 Mining and Metallurgy Institute Bor, 19210 Bor, Serbia; 4 Department of Biology and Ecology, Faculty of Science, University of Kragujevac, 34000 Kragujevac, Serbia; 5 Department of Physiology, Faculty of Medical Sciences, University of Kragujevac, 34000 Kragujevac, Serbia; 6 Institute for Information Technologies Kragujevac, Department of Science, University of Kragujevac, 34000 Kragujevac, Serbia

**Keywords:** meadowsweet, *Salvia*, silver nanoparticles, antioxidant activity, antimicrobial activity, cell viability

## Abstract

The study’s objective was to obtain silver nanoparticles (SVAgNP and FUAgNP) using aqueous extracts of *Salvia verticillata* and *Filipendula ulmaria*. The optimal conditions for nanoparticle synthesis were determined and obtained; nanoparticles were then characterized using UV-Vis, Fourier-transform infrared spectroscopy (FTIR), X-ray powder diffraction (XRD), Dynamic Light Scattering (DLS), Scanning Electron Microscopy with Energy Dispersive Spectroscopy (SEM/EDS). SVAgNP and FUAgNP possessed a crystalline structure with 48.42% and 60.41% silver weight, respectively. The highest percentage of nanoparticles in the solution had a diameter between 40 and 70 nm. In DPPH˙ and ABTS˙^+^ methods, FUAgNP (IC_50_ 15.82 and 59.85 µg/mL, respectively) demonstrated a higher antioxidant capacity than SVAgNP (IC_50_ 73.47 and 79.49 µg/mL, respectively). Obtained nanoparticles also showed pronounced antibacterial activity (MIC ˂ 39.1 µg/mL for most of the tested bacteria), as well as high biocompatibility with the human fibroblast cell line MRC-5 and significant cytotoxicity on some cancer cell lines, especially on the human colon cancer HCT-116 cells (IC_50_ 31.50 and 66.51 µg/mL for SVAgNP and FUAgNP, respectively). The nanoparticles demonstrated high catalytic effectiveness in degrading Congo red dye with NaBH_4_. The results showed a rapid and low-cost methodology for the synthesis of AgNPs using *S. verticillata* and *F. ulmaria* with promising biological potential.

## 1. Introduction

One of the most prominent research fields in recent years is nanotechnology. Since the 1970s, when the development of nanoscience began, this branch of science has been attracting a lot of attention from scientists and taking on ever-greater dimensions of research [1]. Nanotechnology implies a multidisciplinary type of research related to particles whose size is expressed in parts per billion (10^9^), hence from 1 to 100 nm [2]. There are different forms of nanostructures, such as nanoparticles (NPs), nanotubes, and nanopores. All of them have quite interesting properties which, depending on the method of synthesis and purpose, can be used in various segments of the industry [3]. Metal NPs draw attention to their significant properties, particularly as catalysts, biosensors, drug delivery, bio-imaging, the textile industry, supplements in the pharmaceutical and food industries, etc. [4]. The modes of metal NP synthesis can be diverse, ranging from physical to chemical methods. These types of synthesis required the consumption of a considerable number of resources and expensive equipment, as well as the use of harmful chemicals that can lead to environmental challenges and toxicities. In that sense, researchers were focused on finding the ideal solution for NP synthesis, which resulted in a biological approach using green technologies. A biological way of dealing with this subject includes reducing and stabilizing agents of biological origin, i.e., medicinal plants, microorganisms, and isolated natural compounds [5]. The “greening” of this process led to the cessation of the use of large quantities of harmful organic substances, an increase in efficiency, a reduction in toxicity and eco-toxicity, and a reduction in the process price in comparison to previous physical and chemical methodologies [2,6]. Great success in the synthesis of metal and metal oxide nanoparticles that exhibited different biological and catalytic activities, such as silver, ZnO and MgO nanoparticles, was achieved using plant extracts, marine macroalgae (*Ulva fasciata* Delile), bacteria *Pseudomonas aeruginosa*, and brown algae (*Cystoseira crinita*) [7,8,9,10].

Some of the most studied and frequently synthesized metallic NPs are silver nanoparticles (AgNPs). They show significantly different properties when compared to the starting substances, primarily due to the increased ratio between the volume and the surface, which increases their biological activity [11]. Besides their high electrical conductivity and optical effects, AgNPs have been recognized for their distinctive antimicrobial properties [2,5,6]. During their green synthesis, many compounds from natural origin may serve as reducing and capping agents, as well as stabilizers of NPs. Besides primary biomolecules, such as polysaccharides and proteins, many water-soluble products of secondary plant metabolism can be applied in AgNP synthesis. Some of the phytochemicals used are alkaloids and terpenoids, but also polyphenolic compounds such as flavonoids, phenolic acids, and catechins [3,5]. The most common type of silver nanoparticle synthesis is with the help of plant extracts. In this sense, it must be borne in mind that plant extracts are actually a mixture of a large number of various compounds that differ significantly from each other; there is also a disparity in the relationship between the compounds that act as reductants or as stabilizers [11]. That is why it is necessary to define suitable species for the plant-mediated synthesis of AgNPs and properly optimize the process conditions.

*Filipendula ulmaria* (L.) Maxim., known as meadowsweet, belongs to the family Rosaceae (genus *Filipendula* Mill.). It is well known for its antioxidant and anti-inflammatory properties [12,13,14,15], as well as cytotoxic effects [16], but it also possesses antimicrobial effects [17]. Previous investigations showed quite a rich phytochemical composition of the extracts of this plant. The ones that are mainly present are phenolic compounds, primarily compounds from the group of phenolic acids (salicylic acid, gallic acid), flavonoids (quercetin, catechins, hyperoside, spiraeoside), and tannins (tellimagrandins and rugosins) [18,19,20]. Regarding *Salvia verticillata* L. (lilac sage), which belongs to the genus *Salvia* (Lamiaceae), a limited number of investigations have been completed so far. Generally, the extracts of this plant showed notable antioxidant potential [21,22,23] and interesting anti-inflammatory properties [24]. The chemical composition of *S. verticillata* extracts is characterized by compounds indicative of the genus *Salvia*, such as danshensu, rosmarinic acid, salvianolic acids, and derivatives of luteolin and apigenin [23,25]. Our previous investigations of both plant extracts suggested significant antioxidant activity but moderate antimicrobial potential [14,23].

This research aimed to develop and optimize an efficient method of AgNP synthesis using extracts of *S. verticilata* and *F. ulmaria* aerial parts (SVAgNPs and FUAgNPs) in order to improve biological properties and define new ways of use. The characterization of obtained AgNPs was completed by several analytical methods. Biological properties of SV- and FUAgNPs were evaluated using antioxidant, antimicrobial, and cell viability assays, and catalytic effects were monitored as well.

## 2. Results and Discussion

### 2.1. Optimization Conditions and Synthesis of SVAgNP and FUAgNP

The synthesis of AgNPs obtained using the *S. verticillata* and *F. ulmaria* aqueous extracts was visually monitored by a change in the color of the solution from light yellow to an intense brown color. The nanoparticle formation was also tracked spectrophotometrically by recording the UV-Vis absorption spectrum from 800 to 300 nm. The development of a strong peak in the absorption spectra at about 400 nm verified a presence of AgNPs in the solution. The phenomenon known as Surface Plasmon Resonance (SPR) appears due to the interaction of electromagnetic radiation with the surface of the nanoparticles, which causes a change in color.

**Figure 1:**
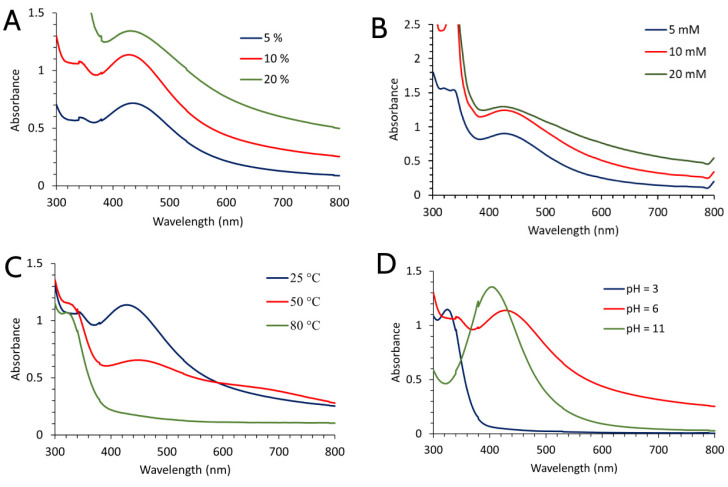
UV-Vis absorption spectra of AgNPs synthesized using *S. verticillata* aqueous extract depending on the application of different synthesis conditions (extract concentration (**A**), concentration of AgNO_3_ (**B**), temperature (**C**), and pH of extract solution (**D**)).

**Figure 2:**
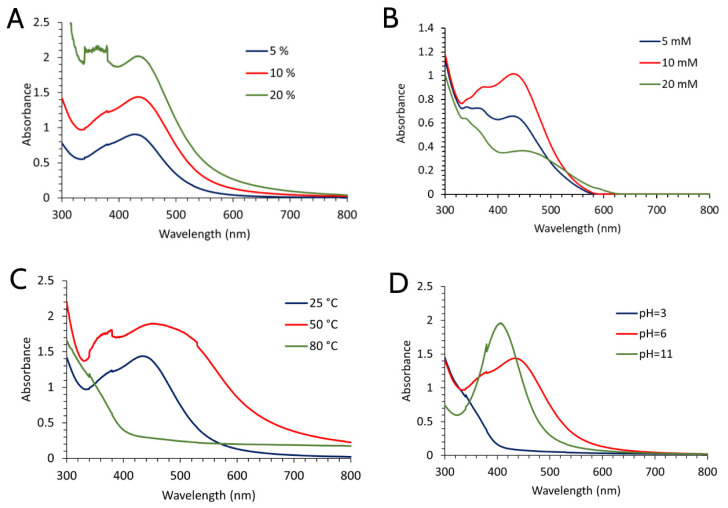
UV-Vis absorption spectra of AgNPs synthesized using *F. ulmaria* aqueous extract depending on the application of different synthesis conditions (extract concentration (**A**), concentration of AgNO_3_ (**B**), temperature (**C**), and pH of extract solution (**D**)).

To achieve the most optimal synthesis conditions, the effects of changes in extract concentration, temperature, and pH values were examined while the salt concentration (AgNO_3_) used for the synthesis of both types of nanoparticles was 10 mM as can be seen from Figure 1B and Figure 2B. In both types of nanoparticles, higher absorption peaks were obtained when extract concentrations were increased (20%) (Figure 1A and Figure 2A). However, their width was significantly higher, which indicated the presence of a wide range of nanoparticle sizes. While the height of peaks provides information about the concentration of nanoparticles, their width offers information about their size distribution. For example, the lower size range of nanoparticles in the solution corresponds to the narrower peak. Additionally, their size diminishes because of the shifting of the peak to shorter wavelengths. For this reason, 10% extract solutions of both extracts used were the most optimal for the synthesis of larger quantities of nanoparticles. It is observed that the absorption peaks decrease as temperature rises (Figure 1C), indicating that a detrimental impact on SVAgNP production may result from the temperature rise. The effect of temperature on the synthesis of FUAgNP was somewhat different; the maximum absorption was reached at 50 °C (Figure 2C). However, the peak obtained at 50 °C was not sharp and there was a possibility of the presence of large particles that could easily initiate agglomeration. The peak that appeared at 25 °C had a more regular shape, while at 80 °C there was complete agglomeration and deposition of particles. Contrary to this, Sathiskumar et al. [26] observed a rise in surface plasmon resonance as temperature rises, supporting the idea that temperature and nanoparticle production are positively correlated. According to results of SVAgNP and FUAgNP synthesis conditions, optimal temperature for plant-mediated synthesis of AgNPs may vary depending on the applied plant material. It has long been known that the presence of H_3_O^+^ and OH^-^ ions can affect many chemical and biochemical processes. In this case, the presence of OH^-^ ions had an activating effect on the synthesis of nanoparticles, while H_3_O^+^ ions had an inhibiting effect. It is assumed that OH^-^ ions activate the hydroxyl groups on the phenolic compounds and thus enable the rapid reduction in silver ions. In this way, a quick reaction and greater stabilization of nanoparticles are also enabled [27]. Accordingly, the influence of pH on the synthesis of both types of nanoparticles had the same effect: at pH 11 the synthesis was the best, while at pH 3 practically no nanoparticles were formed (Figure 1D and Figure 2D).

**Figure 3:**
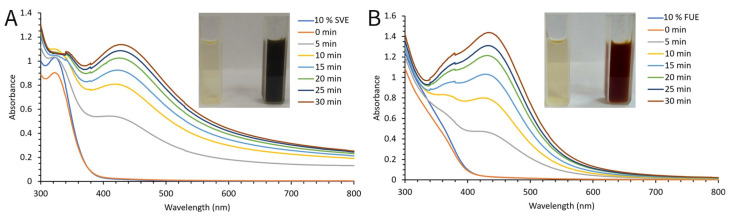
Time-dependent UV-Vis absorption spectra of SVAgNP (**A**) and FUAgNP (**B**) synthesis under optimal conditions.

For physical, chemical, and biological research, larger quantities of both types of nanoparticles were synthesized by combining the most favorable conditions, as well as being guided by previous knowledge about this environmentally acceptable method of synthesis (Figure 3A,B). The absorption maximum’s development stopped 30 min after AgNO_3_ addition, signaling the completion of the reaction. A sharper and more prominent peak was achieved by synthesis using *F. ulmaria* extract, indicating the presence of compounds with better reducing and capping properties.

Earlier research published by Katanić Stanković et al. [23] reported that rosmarinic acid as phenolic acid and luteolin-*O*-glycoside as flavonoid, were most abundant in the methanol extract of the *S. verticilata* aerial part. Since flavonoid compounds have reducing properties, they reduce Ag^+^ ions to Ag^0^, changing their enol to keto form, and as such, they are incorporated into the structure of the resulting nanoparticles. In contrast to *S. verticillata* methanolic extract, which contains various phenolic acids, *F. ulmaria* extract contains a wide range of flavonoids, including catechin, epicatechin, and rutin [14]. The phytochemistry of the aqueous extracts of these plant species and the role of compounds responsible for the synthesis and stabilization of the AgNPs will be discussed in more detail in the next sections for FTIR, total phenolic, and flavonoid results interpretation.

### 2.2. Fourier-Transform Infrared Spectroscopy (FTIR)

**Figure 4:**
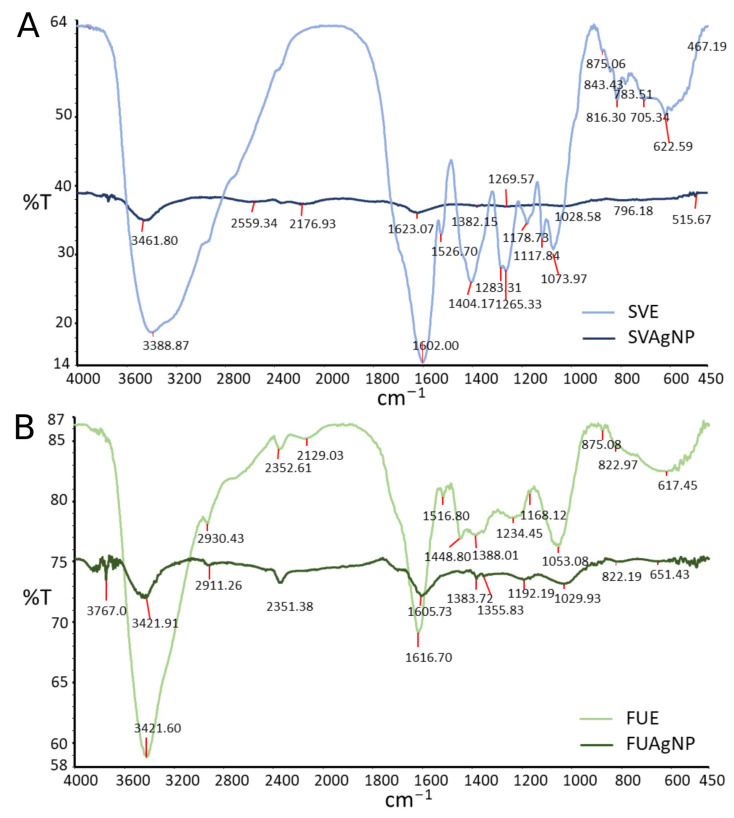
Fourier-transform infrared spectra (FTIR) of *S. verticillata* (**A**) and *F. ulmaria* (**B**) extracts and corresponding AgNPs.

FTIR spectroscopy analysis was used to determine the possible functional groups of compounds present in SVE and FUE that participate in the synthesis of NPs. The comparative FTIR spectra of SVE and FUE, and their derived AgNP, are shown in Figure 4. The spectral data showed broad bands at 3388 and 3421 cm^−1^ in the SVE and FUE IR spectra of extracts, respectively. These are typical IR bends, but attenuated and/or shifted, related to the stretching vibration of -OH groups found in alcohols and phenols; hydrogen-bonded groups were also observed in the SVAgNP and the FUAgNP at 3461 and 3421 cm^−1^, respectively. Characteristic bands for aliphatic C-H stretching vibration were observed at 2930 cm^−1^ and 2911 cm^−1^ for FUE and its corresponding AgNPs, respectively, while in SVE this band was not observed due to the wide range of the band attributed to the stretching vibration of -OH groups. Intensive bands at 1602 and 1616 cm^−1^ were observed in the FTIR spectra of SVE and FUE, respectively. This broad band in the 1600 and 1700 cm^−1^ region could be attributed to the C=C stretching vibration of aromatic rings, as well as the C=O group of amides, esters, or C=O group on the C ring of flavones [28,29,30,31]. These intensive bands were also noticed at similar positions in the SVAgNP and the FUAgNP FTIR spectra (1623 and 1605 cm^−1^, respectively), but attenuated. The bands at 1404 and 1448 cm^−1^ in SVE and FUE spectra, may be assigned to C-O bending vibrations of carboxylic acids or esters, O-H bending vibration of alcohol and phenols, and bending vibration of N-H in amides. The medium broad bands at 1265 and 1073 cm^−1^ are found in the SVE specter and in FUE at 1234 and 1035 cm^−1^; this is also the C–O stretching mode of the alcohols, carboxylic acids, esters, ethers, carbohydrates residues, and flavonoids (C-O-C group) [28,32,33,34].

The same bands that belong to the phytochemicals’ functional groups such as the phenolic group, aromatic ring, C=O, ester, or amid groups, as well as the C–O–C groups of flavonoids in AgNP spectra at similar positions as in extract FTIR spectra, suggest the role of these compounds in the stabilization of formed AgNPs. Except for a reduction in some peak intensity in AgNP spectra, peaks found at 1404 cm^−1^ for SVE and 1448 cm^−1^ for FUE in FTIR spectra associated with C–O and C–OH bending vibrations, were not found in corresponding AgNP spectra. This may be the consequence of involving some –OH or C–O bearing groups in the reduction reaction of Ag^+^ to Ag^0^ and the chemical change of this functional group.

### 2.3. SEM/EDX Analysis

**Figure 5:**
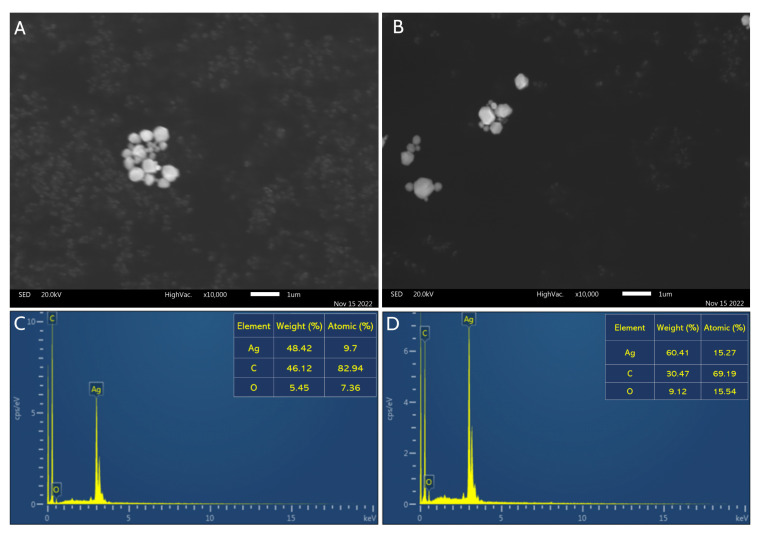
SEM image of synthesized SVAgNP (**A**) and FUAgNP (**B**) with corresponding EDX spectra ((**C**,**D**), respectively).

Figure 5 displays the results of the SEM and EDX studies for SVAgNP and FUAgNP, respectively. While SEM analysis was used for more information about nanoparticle surfaces’ structural morphology, EDX analysis gives us details about elements presented in SVAgNP and FUAgNP. The results show that the nanoparticles obtained possess a spherical form with less degree of agglomeration. Pirtarighat et al. [35] synthesized AgNPs using *Salvia spinosa* and also obtained spherical nanoparticles. Figure 5A,B clearly show the presence of small nanoparticles. However, the presence of larger lumps can be explained by the agglomeration that occurs as a result of drying the sample, which is necessary for this technique. Due to their propensity to aggregate upon drying, DLS analysis was used to precisely determine the size of nanoparticles from the solution. The absorption of AgNPs results in a strong signal with a peak at 3 KeV [36], and this signal was seen in the EDX spectra of SVAgNP and FUAgNP (Figure 5C,D). The presence of organic components in the structure of nanoparticles was confirmed by EDX analysis, which showed that, in addition to silver atoms, SVAgNP and FUAgNP are mostly composed of carbon and oxygen from plant phytochemicals on their surface. The presence of some other elements in the FUAgNP structure is explained by the fact that the plant can also absorb some other elements from the soil that can be easily incorporated into the nanoparticle structure [27].

### 2.4. XRPD Analysis

**Figure 6:**
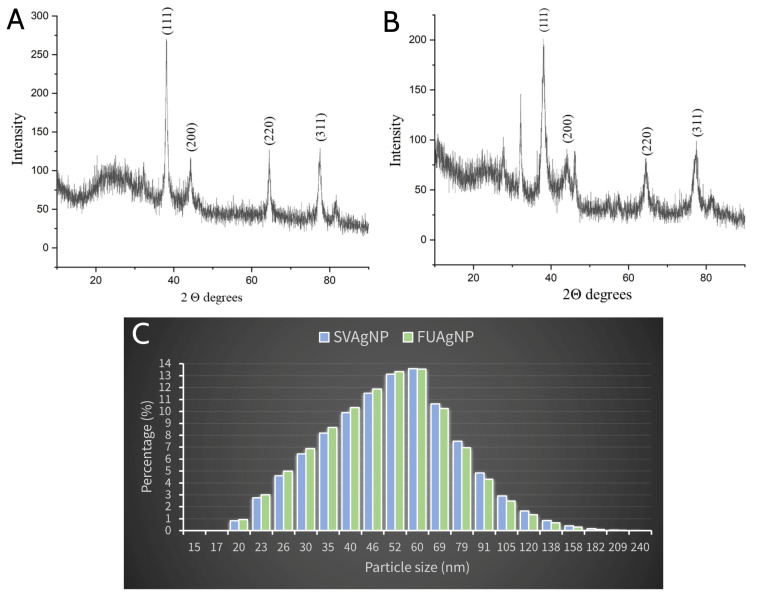
XRPD pattern of synthesized SVAgNP (**A**) and FUAgNP (**B**); DLS analysis of synthesized SVAgNP and FUAgNP (**C**).

Both types of synthesized nanoparticles were subjected to XRPD analysis to confirm their crystalline and nanostructure. The presence of four peaks at 2Θ = 38.07°, 44.24°, 64.52°, and 77.47° in FUAgNPs (Figure 6B) corresponds to the presence of the Bragg reflection planes (111), (200), (220), and (311), thus indicating the presence of FCC (face centered cubic) and crystal structure of FUAgNP. Similar results were obtained by recording SVAgNPs, the peaks located at 2Θ = 37.97°, 44.11°, 64.71°, and 77.39°, corresponding to the presence of the same Bragg reflection planes, i.e., (111), (200), (220), and (311), and confirming the FCC crystal structure of SVAgNP (Figure 6A) [37,38]. The XRD peak position, height, and width determine the nanocrystalline nature and purity of the nanoparticles. Sharp peaks in the X-ray diffractogram of the biosynthesized AgNPs indicate that the synthesized AgNPs possess crystal structures.

### 2.5. Dynamic Light Scattering (DLS) Analysis

DLS analysis examines the hydrodynamic diameter of AgNPs in solution, which takes into account both molecules and ions attached to their surface in solution. Numerous studies have suggested the value of hydrodynamic diameter in determining the size of nanoparticles and enhancing their functionality in biological experiments [39]. Based on the results of the DLS analysis, it can be observed that both types of synthesized AgNPs have very similar sizes (Figure 6C). However, based on the size distribution, it can be concluded that a larger percentage of the AgNPs synthesized using the *F. ulmaria* extract are somewhat smaller, which coincides with the previous claims about the position of the absorption peak in the spectrum.

Both types of nanoparticles ranged in size from 20 to 209 nm, while the highest percentage of the nanoparticles were in the size range from 40 to 70 nm. The difference in the size of the nanoparticles went up to 189 nm, which indicates a wider range of different AgNP sizes. A higher percentage of nanoparticles with size from 20 to 50 nm and lower percentage of FUAgNPs larger than 50 nm in diameter was observed in the solution when compared with SVAgNP. These results clearly indicate that the application of FUE in AgNPs may produce particles of smaller dimensions. The size and form of the silver nanoparticle synthesized using plants are influenced by a wide range of variables. Many published studies confirm that the pH value is a parameter that can be used to control the size of nanoparticles. It was observed that broad absorption peaks are obtained in an acidic environment, and that as the size of the nanoparticles decreases when the pH value increases, the absorption increases, and a narrow peak with a more uniform size distribution is obtained. So, with an alkaline medium, the stability of nanoparticles and the formation of colloids increase, while there is a decreasing preference towards their agglomeration [40]. Sathishkumar et al. [41] have proposed a theory according to which at higher pH values, the availability of many functional groups enables the rapid binding of Ag (I), forming a large number of nanoparticles of smaller dimensions.

### 2.6. Phenolic Content in Extracts and Antioxidant Activity of Synthesized Nanoparticles

**Figure 7:**
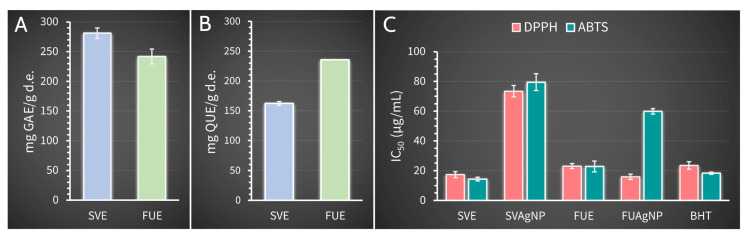
Total phenolic (**A**) and flavonoid (**B**) content of *S. verticillata* and *F. ulmaria* aqueous extracts and the antioxidant activity (**C**) of synthesized SVAgNP and FUAgNP and corresponding extracts.

Plants, especially medicinal plants, are recognized as a valuable source of non-enzymatic exogenous antioxidants for humans in the fight against reactive species and oxidative stress [42]. These plants’ antioxidants, in addition to participating in the formation of nanoparticles due to their ability to reduce metal ions, are attached to the surface of nanoparticles, stabilize them, and provide their antioxidant properties [43]. Both aqueous extracts used for nanoparticle synthesis in this study possessed high total phenolic and flavonoid contents (Figure 7A,B), indicating their high potential to reduce Ag^+^ ions and act as a capping agent that binds to the surface of the formed nanoparticles, and showing the strong effect on the final size of the synthesized AgNPs. Although the extracts used were similar in the total phenolic contents, differences were observed in the flavonoid content of the extracts: FUE contained a significantly higher number of total flavonoids when compared with SVE. The higher flavonoid contents, as well as differences in the structure of phenolic compounds in SVE and FUE, may be the cause for observed differences in diameter size (Figure 5C) and biological activities of synthesized SVAgNP and FUAgNP. The AgNPs obtained showed a high antioxidant potential determined in the DPPH and ABTS^·+^ methods (Figure 7C). However, SVE and FUE revealed higher DPPH and ABTS^·+^ scavenger capacity than corresponding AgNPs synthesized using these aqueous extracts, except for FUAgNP in the DPPH method. The higher antioxidant potential in both methods showed FUAgNP with IC_50_ values of 15.82 and 59.85 µg/mL for DPPH and ABTS^·+^ scavenger activity, respectively, compared with SVAgNP (IC_50_ of 73.47 and 79.49 µg/mL, respectively). The opposite trend was observed among extracts; SVE was more effective in the neutralization of free radicals than FUE. These results indicate that the antioxidant potential of synthesized AgNPs does not depend exclusively on the antioxidant activity of secondary biomolecules present in the extract used for their synthesis. Most likely, the antioxidant activity of nanoparticles synthesized by plants depends on different factors such as the antioxidant potential of compounds present on the surface of the AgNPs, the interaction of these compounds with AgNPs, the potential of antioxidants from the extract to bind to nanoparticles, and probably also on the size of the obtained AgNPs. The better antioxidant potential of FUAgNP when compared with the SVAgNP may be related to the slightly smaller particle diameter of the FUAgNP; in fact, a higher percentage of particle size between 20 and 60 nm and lower percentage of particle size between 60 and 180 nm were observed in solution for FUAgNP when compared to SVAgNP (Figure 6C). Moges and Goud [44] concluded that phenolic compounds and flavonoid contents of AgNPs synthesized using *Hippophae salicifolia* leaves and berries have a great impact on nanoparticle size and antioxidant properties. Still, in this study, by comparing the antioxidant activity of AgNPs of different sizes obtained using extracts from the same plant material, but using different extraction solvents, it was shown that the AgNPs larger in size were more active than the AgNPs smaller in average particle size. According to this finding and the results of the antioxidant activity of SVAgNP and FUAgNP, it can be assumed that flavonoids, which are able to bind to nanoparticles present in FUA, are responsible for the great antioxidant activity of FUAgNP.

Previously published results suggested that most AgNPs synthesized using plant extracts have higher antiradical activity than their corresponding extracts, which is opposite to the results obtained in this study [44,45,46]. Only FUAgNP displayed higher antioxidant potential in DPPH methods in comparison with the antioxidant activity of both extracts used for nanoparticle preparation. Although SVE and FUE had a strong antioxidant effect, there is the possibility that, during the synthesis of nanoparticles, the antioxidants present in the extracts do not completely bind to their surface, or certain antioxidants may be chemically modified in reactions with Ag^+^ or AgNPs. Aqueous extracts, SVE and FUE, used in this study demonstrate strong antioxidant potential and high phenolic and flavonoid content. These results were comparable, with some differences, to those obtained in previous publications for methanolic extracts of *S. verticillata* and *F. ulmaria* aerial parts. According to Katanić Stanković et al. [23], *S. verticillate* methanolic extract possessed a lower quantity of total phenolic compounds (175.6 mg GA/g of dry extract) and higher flavonoid content (244.4 mg QU/g of dry extract) than SVE (281.20 mg GA/g of dry extract and 162.54 mg QU/g of dry extract, respectively). The same research also showed that *S. verticillata* methanolic extract had lower DPPH and ABTS radical scavenger activity than SVE [23]. The aqueous extract, FUE, used for the synthesis of FUAgNP, contained similar total phenolic content (241.86 mg GA/g of dry extract) and dramatically higher flavonoid content (235.70 mg QU/g of dry extract) when compared with methanolic extracts of *F. ulmaria* aerial parts (249.53 mg GA/g of dry extract and 45.47 mg QU/g of dry extract, respectively) [14]. Similar antioxidant activity of *F. ulmaria* methanolic extract and FUE was also observed [14]. The high phenolic content and the high antioxidant capacity of the extracts of these two plant species provided high antioxidant potential of synthesized SVAgNP and FUAgNP, when compared with some recently published research about the antioxidant activity of dealing with *Salvia* species-mediated synthesized AgNPs. The results for the antioxidant activity of SVAgNP are comparable to those obtained for AgNPs synthesized using *Salvia officinalis* leaf aqueous extract (IC_50_ 79 µg/mL for DPPH method) [47]. In the study conducted by Siakavella et al. [48], AgNPs synthesized using *S. officinalis* extract demonstrated lower antioxidant potential with an IC_50_ of 770 µg/mL when compared with the antioxidant activity of SVAgNP in the DPPH method and AgNPs studied in the aforementioned research. The high antioxidant potential of AgNPs synthesized using *S. officinalis* extracts [49,50], as well as *S. coccinea* [51], *S. aethiopis* [52], and *S. hispanica* [53] extracts, was also confirmed in different studies. However, there is no literature data about the synthesis and antioxidant potential of AgNPs synthesized using plants from *Filipendula* genus.

### 2.7. Antimicrobial Activity

The results for the antimicrobial activity of synthesized nanoparticles are shown in Table 1. Both silver nanoparticles, SVAgNP and FUAgNP, manifested high antibacterial potential for tested bacterial species. According to obtained MIC values, SVAgNP and FUAgNP inhibited the bacterial growth of most bacterial species at the same concentrations. However, FUAgNP with MIC values below the lowest tested concentration (39.1 µg/mL) for seven bacterial species was more effective in the inhibition of bacterial growth for four bacterial species when compared with SVAgNP. The least sensitive bacterial species to the effect of both synthesized nanoparticles are *E. coli*, *M. lysodeikticus,* and *S. epidermidis* with MIC values of 156.2, 625.0, or 2500 µg/mL. The MICs of SVAgNP and FUAgNP were below 100 µg/mL for all other bacterial species, while antibiotic ciprofloxacin demonstrated antibacterial activity against examined bacterial species with MIC values below 20 µg/mL.

The fungal species used in this study were less sensitive to SVAgNP and FUAgNP effects (Table 1). In contrast to the antibacterial activity of studied nanoparticles, where FUAgNP showed a slightly better effect, SVAgNP and FUAgNP displayed approximately the same level of antifungal activity with the same MIC values except for *T. lougibrachiatum*. The most sensitive fungal species to SVAgNP and FUAgNP were two studied *Penicillium* molds (MIC ˂ 78.1 µg/mL for both synthesized NPs), *T. lougibrachiatum* (MIC 312.5 and ˂78.1 µg/mL, respectively), and yeast *C. albicans* (MIC 312.5 µg/mL for both synthesized NPs). *A. brasiliensis, F. oxysporum,* and *A. alternata* were resistant to SVAgNP and FUAgNP effects when they were applied at a concentration up to 10 × 10^3^ µg/mL. Clotrimazole showed antifungal activity with MIC values in the range <0.3125–40 μg/mL against examined fungal species.

Some differences in the antibacterial activity of SVAgNP and FUAgNP may be attributed to the different particle sizes or phytochemicals on their surface. It was observed that nanoparticles of smaller dimensions pass through the bacterial cell membrane more easily and exert an antibacterial effect inside the cell [54]. Differences in the chemical composition of extracts used for AgNP synthesis and, consequently, the presence of antimicrobial phytocompounds from those extracts on the surface of AgNPs, can also lead to differences in their antimicrobial activity [55]. The results obtained in previously published papers demonstrated that AgNPs synthesized using *S. verticillata* and *F. ulmaria* extracts possessed significantly better antimicrobial properties than their methanolic extracts. Katanić Stanković et al. (2020) found that methanolic extracts of the *S. verticillata* aerial part had a low antimicrobial activity with MIC values of 20 × 10^3^ µg/mL for most of the studied bacterial species, and higher than 20 × 10^3^ µg/mL for fungal species [23]. *F. ulmaria* aerial part methanolic extract with MICs of 5 × 10^3^ µg/mL for most tested bacterial species was less effective than the FUAgNP, except for *E. coli* [14]. In comparison with some recently published results for the antibacterial activity of AgNPs synthesized using plant extracts [27,56,57], SVAgNP and FUAgNP possessed similar or higher antibacterial potential. AgNPs synthesized using *Salvia* species are proven as good antibacterial materials, displaying higher antimicrobial potential than corresponding plant extracts used for their preparations [49,56]. According to the results obtained for inhibition of *S. epidermidis* (MIC 187.5 µg/mL) and *P. aeruginosa* (MIC 375 µg/mL) growth by AgNPs synthesized using *Salvia africana-lutea* extract, SVAgNP inhibited the growth of these two bacterial species in lower concentrations (156.2 and 78.1 μg/mL, respectively) [56]. 

Several mechanisms of antimicrobial activity in metal nanoparticles have been proposed, including disruption of microbial cell membrane nanoparticles and increase in its permeability, accumulation of metal ions in the cell, and generation of reactive oxidative species (ROS) in the cell. All these three possible mechanisms are linked, and may affect enzymatic reaction of the cell, liberation of an intracellular component outside the bacterial cell, respiratory chain reactions, and/or block replication of DNA reacting with nitrogen bases [58,59,60].

### 2.8. Cytotoxic Activity/The Effects on Cell Viability

**Figure 8:**
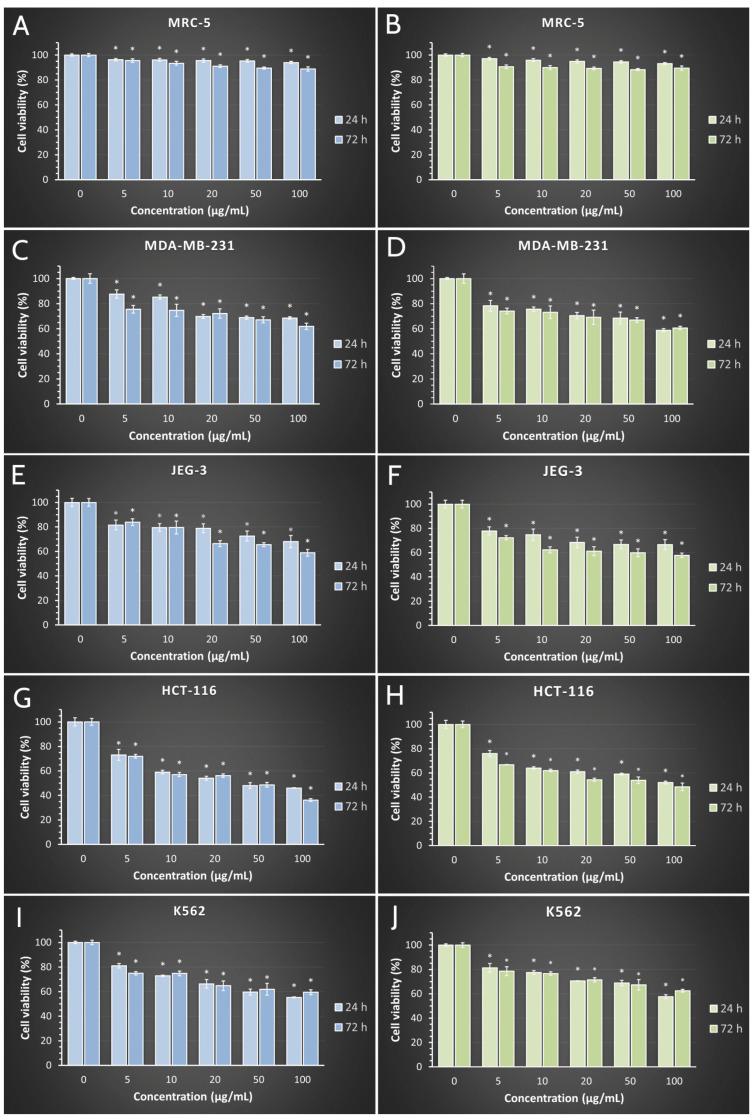
Effects of synthesized SVAgNP (**A**,**C**,**E**,**G**,**I**, blue bars) and FUAgNP (**B**,**D**,**F**,**H**,**J**, green bars) on normal (MRC-5) and cancer (MDA-MB-231, JEG-3, HCT-116, and K562) cell viability after 24 h and 72 h of treatment. Results are presented as the mean of three independent experiments ± standard error; * *p* < 0.05 relative to control.

This study investigated the effect of five different concentrations (5 to 100 µg/mL) of SVAgNP and FUAgNP on the normal human lung fibroblast cell line MRC-5, the human breast cancer cell line MDA-MB-231, the human placental choriocarcinoma JEG-3 cell line, the human colon cancer HCT-116 cell line, and the human chronic myelogenous leukemia K562 cell line. Cells were exposed to treatment for 24 h (short-term) and 72 h (long-term). The effect of investigated treatments with SVAgNP and FUAgNP on the viability of the normal human fibroblast cell line MRC-5 during 24 h and 72 h was assessed to estimate the biocompatibility of the tested particles. The results (Figure 8) show that the maximal inhibition of viability of cultivated MRC-5 cells was 88.88% when compared to non-treated cells in a concentration of 100 µg/mL. Accordingly, this analysis demonstrated that the tested nanoparticles did not exert a non-specific toxic effect. The selectivity obtained in our study is in concordance with previous experiments regarding targeted effects of AgNPs in cancer cells [61,62,63].

The results of cell viability in human cancer cell lines after 24 and 72 h of exposure to investigated treatments are presented in Figure 6. The results obtained showed that all applied treatments after 24 h caused a decrease in cell viability in all four tested cell lines. IC_50_ values below the maximal tested of 100 µg/mL were obtained for SVAgNP in both the 24 h (44.62 µg/mL) and 72 h (31.50 µg/mL) treatments in the HCT-116 cell line, while for FUAgNP the IC_50_ values were detected in 72 h treatments (66.51 µg/mL). These data imply that HCT-116 cells are the most sensitive to the treatment, suggesting that the AgNPs tested in our study could be most effectively used for the treatment of colon cancer, which also has been indicated in the literature [64]. For all five tested concentrations of both nanoparticles, the viability of exposed cancer cells was significantly decreased compared to control cells, and multifold lower when compared to non-cancer human lung fibroblast cells MRC-5. In addition, cell viability after 72 h was further decreased when compared to short-term treatment, suggesting a time dependent effect. 

Previously published research demonstrated similar results for cytotoxicity of AgNPs synthesized using *Salvia* sp. extracts. The synthesized AgNPs using three *Salvia* species extracts, in the previously published study, showed anticancer activity against the A549 human lung cancer cells with IC_50_ values of approximately 400 µg/mL [65], AgNPs synthesized using *Salvia officinalis* extract displayed low cytotoxicity on HeLa cells [66], while in another study, *S. officinalis* synthesized AgNPs reduced viability of different malignant oral cell lines with IC_50_ values between 74 and 180 µg/mL [47].

### 2.9. Catalytic Potential of SVAgNP and FUAgNP

For the first preliminary examination of the potential catalytic activity of some material or molecule, the method of degradation most often used is the Congo red. Congo red is obtained by azo coupling of bis diazonium derivatives of benzidine with naphthenic acid. The complex aromatic structure makes this molecule stable against oxidizing agents and resistant to biodegradation, which results in its long retention in the environment [67]. Before learning about its carcinogenicity and high environmental toxicity, this synthetic dye was used in many branches of industry, such as paper, textile, and rubber production [68]. Interestingly, Congo red is still used in medicine for amyloid detection, in vivo visualization, to stabilize partially folded and native protein monomers, for the specificity of staining by establishing bonding with fibril proteins and for use in designing imaging probes [69]. However, in underdeveloped countries, still larger quantities of Congo red end up in watercourses. Today, several different treatments are used in the purification of wastewater, membrane separation, absorption, photocatalysis, and ozonization [70]. However, compared to the mentioned techniques, catalytic reduction is preferred since the reduction products of Congo red find uses in a variety of sectors [69]. In recent years, there have been more and more attempts to synthesize nanocatalysts that will easily and quickly degrade synthetic dyes into safer products for the living world. The efficiency of SVAgNP and FUAgNP in the degradation of Congo red at room temperature was investigated. The catalytic degradation reactions of Congo red were monitored spectrophotometrically by decreasing the absorption peak at 497 nm. The possible mechanism of this degradation is explained by the role of AgNPs in the transfer of electrons from NaBH_4_ to Congo red, which results in its reduction [71]. The degradation of CR was investigated using NaBH_4_ and synthesized AgNPs as catalysts. In this reaction, AgNPs were used for the transfer of electrons from electron donor BH_4_^‾^ to CR which is an electron acceptor. This transfer reduces activating energy for CR reduction and stabilizes the system [53]. AgNPs also represent a suitable surface for attaching both reactants, CR and BH_4_^‾^, and allowing the reaction to take place [34]. Fauda et al. [58] have shown that light can also initiate silver nanoparticle catalysis. The light excites the electrons on the surface of the nanoparticles, during which they move from the valence band to the conduction band. Afterwards, they interact with water molecules and form free radicals. Free radicals then react with synthetic dyes and degrade them.

**Figure 9:**
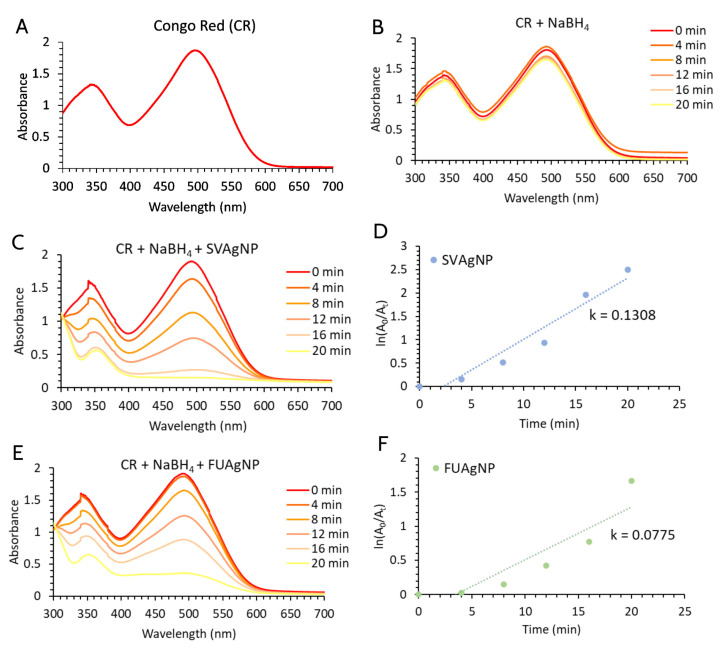
(**A**) UV–Vis spectra of the CR dye; (**B**) Degradation of CR in the presence of NaBH_4_; The catalytic activity of SVAgNP (**C**) and FUAgNP (**E**) on CR degradation in the presence of NaBH_4_; Degradation kinetics of CR in the presence of SVAgNP (**D**) and FUAgNP (**F**).

The Congo red absorption spectra are shown in Figure 9A, while Figure 9B shows the degradation of Congo red by NaBH_4_ in the absence of nanoparticles, which does not actually occur. The preliminary study with different concentrations of AgNPs showed that the concentration of AgNPs of 100 μg/mL is the most optimal for recording the kinetics of the reaction. The use of higher concentrations of AgNPs as catalysts in CR degradation caused a very quick reaction; the color of the concentrated AgNP solution also interfered with the color of CR. At low concentrations, the reaction proceeded quite slowly. The catalytic activities of SVAgNP and FUAgNP are presented in Figure 9C,E, and both types of nanoparticles showed a high potential for the catalytic degradation of CR in the presence of NaBH_4_. The potential overlapping of absorption peaks originating from AgNPs and Congo red was avoided using a low concentration of nanoparticles, which also proved effective. Over time, Congo red’s absorbance gradually declined to the point where the solution was colorless, and the UV-Vis absorption peak had almost completely disappeared. The catalytic rate constant (k) for SVAgNP (Figure 7D) (k = 0.1308) is significantly higher compared to the rate constant calculated for degradation by FUAgNP (Figure 9F) (k = 0.0775). It was also noticed that in the absence of light, this process is much slower. The degradation of Congo red using AgNPs synthesized by *Salvia officinalis* was also researched by Albeladi et al. [38]. According to the results from that study, when the concentration of nanoparticles and NaBH_4_ increases, the rate of degradation also increases, owing to the increased surface area of the nanoparticles or to the provision of more electrons from the donor (BH_4_^‾^). It was also observed that the highest degree of degradation was achieved at pH 7. The degradation of extremely hazardous organic compounds and damaging azo dyes can be enabled by the phytochemical-based production of nanoparticles, such as SVAgNP and FUAgNP, which offers a practical, economical, and environmentally favorable alternative.

## 3. Materials and Methods

### 3.1. Materials

The chemicals used in this study (Sodium borohydride (NaBH_4_), silver nitrate (AgNO_3_), 2,2’-azino-bis (3-ethylbenzthiazoline-6-sulfonic acid) (ABTS), sodium dodecyl sulfate (SDS), 2,2-diphenyl-1-picrylhydrazyl 1,1-diphenyl-2-picrylhydrazyl radical (DPPH), referent phenolic standards, and methanol) were procured from Sigma-Aldrich Chemicals (Deisenhofen, Germany). Substrates and components (Nutrient agar (NA), Sabouraud dextrose agar (SDA), Müller–Hinton broth (MHB), and Sabouraud dextrose broth (SDB)) for microorganism cultivation and determination of antimicrobial activity are provided from Torlak Institute of Virology, Vaccines, and Sera (Belgrade, Serbia). The resazurin salt was supplied from Acros Organics (Geel, Belgium). Solvents used in high-performance liquid chromatography (HPLC) analyses were obtained from Roth (Karlsruhe, Germany). The following reagents and chemicals were used for cell viability analysis: Dulbecco’s Modified Eagle medium (DMEM), 10% fetal bovine serum (FBS), 0.4% Trypan blue, 0.25%, trypsin-EDTA, dimethyl sulfoxide (DMSO), 3-(4,5-Dimethylthiazol-2-yl)-2,5-diphenyltetrazoliumbromide (MTT), phosphate buffered saline (PBS). All the chemicals and reagents used in this study were of the highest commercially available purity and obtained from Sigma-Aldrich, Inc. (St. Louis, MO, United States).

### 3.2. The Extract Preparation

The plant material was collected as follows: the aerial parts of *F. ulmaria* were collected at Goč Mountain, Serbia, while the aerial parts of *S. verticillata* were collected in Prijevor, near the Ovčar-Kablar Gorge, Serbia. The identification of species was performed at the Herbarium of the Department of Biology and Ecology, Faculty of Science, University of Kragujevac (Kragujevac, Serbia) and the herbal samples were deposited under voucher numbers 112/013 and 125/016, respectively. Plant parts were freed from impurities and placed in a dark, ventilated room to dry. The dry aerial parts of both plants were finely powdered and 10 g of each were measured. The extraction was done according to Srećković et al. [27] using boiling deionized water (100 mL) for 1 h; afterwards the aqueous extracts of *S. verticillata* and *F. ulmaria* (SVE and FUE) were filtered through Macherey-Nagel 85/70 mm filter paper and stored for maximum of one week at 4 °C. The extracts were used in the synthesis of silver nanoparticles.

### 3.3. Green Synthesis of AgNPs

The method used for the synthesis of AgNPs was described by Markus et al. (2017) with some modifications [72]. Silver nitrate (AgNO_3_) was added into the previously diluted stock solutions of the prepared aqueous extracts FUE and SVE (5, 10, and 20% *v*/*v*) to obtain the concentrations of 10 mM AgNO_3_. All reaction mixtures were maintained on a magnetic stirrer at different temperatures (25, 50, and 80 °C) and stirred until AgNPs were formed. Modification of the reaction mixture was done to correspond to three different values of pH (pH 3, 6, and 11) using 1 M HNO_3_ or 1 M NaOH. The first sign of the reaction development was a visual color change from yellow to dark brown, followed by changes in the UV-Vis spectra (UV-Vis spectrophotometer Halo DB-20S, Dynamica GmbH, Dietikon, Switzerland). The suspensions of AgNPs obtained were centrifuged at 12,000 rpm for 10 min and the resulting residue was resuspended in demineralized water, followed by a second centrifuging to obtain nanoparticles that have been precipitated. AgNPs were subjected to a drying process at 40 °C in a hot air oven, and the end product was stored in the refrigerator at 4 °C until further analysis.

### 3.4. Characterization of Synthesized AgNPs

To track the process of AgNP synthesis, but also to characterize the obtained AgNPs, spectrophotometry measures were included at a wavelength range from 300 to 800 nm, with a resolution of 0.5 nm. Some other measures were included too, as previously reported [27]. The crystal structure of AgNPs was evaluated using an X-ray diffractometer (PHILIPS PW 1710) set at a voltage of 40 kV and 30 mA with CuK α radiation of 1.54178 Å. The range was set from 10 to 90° 2 θ with a step of 0.02° and a retention time of 0.25 s at each step [73]. The surface and the elemental composition of synthesized AgNPs, which were prepared in a JOEL FC-TM20 auto coating thickness controller, were determined by a scanning electron microscope (SEM) JOEL JSM IT 300LV with EDS detector (OXFORD Instruments, X-max). The size of obtained NPs was evaluated using dynamic light scattering (DLS) measurements (Mastersizer 2000 from Malvern Panalytical, Malvern WR14 1XZ, UK). Moreover, the tested NPs, as well as both dry plant extracts, were analyzed via Fourier transform infrared (FTIR) spectroscopy.

### 3.5. Determination of Phenolic Compounds and Antioxidant Activity

The content of total phenolic compounds and flavonoids was determined in SVE and FUE using Folin-Ciocalteu reagents for total phenolics and aluminum chloride for flavonoids. The same protocols were applied as described by Srećković et al. [74] for these determinations. The total phenolic content in the extracts was expressed in mg of gallic acid equivalents per g of the dry weight of extracts (mg GAE/g d.e.), while total flavonoid content was expressed in mg of quercetin equivalent per g of the dry weight of extracts (mg QUE/g d.e.).

For the evaluation of the synthesized AgNPs’ biological properties, the primary method was the determination of antioxidant potential towards 2,2-diphenyl-1-picrylhydrazyl (DPPH^·^) [75] and 2,2′-azinobis-(3-ethylbenzothiazoline-6-sulfonic acid) diammonium radical-cation (ABTS^·+^) [75,76].

The ABTS^·+^ reagent solution was prepared according to the method. Several previously defined concentrations of tested AgNPs (0.1 mL, aqueous solutions) were mixed with ABTS^·+^ reagent (0.9 mL) and incubated for 30 min in the dark. The absorbance was read spectrophotometrically at 734 nm.

For the determination of DPPH^·^ scavenging activity, the same volume of AgNP solution (concentration range from 100 to 0.78 µg/mL) was mixed with DPPH^·^ methanolic solution (80 μg/mL). The absorbances of samples were recorded at 517 nm after 30 min of adding the free radical solution to the AgNP solution. The reaction was performed at room temperature and in a dark place.

Butylated hydroxytoluene (BHT) was used as a reference standard in both radical scavenging methods. The spectrophotometric measurements were performed on a UV/Vis double beam spectrophotometer Halo DB-20S (Dynamica GmbH, Dietikon, Switzerland). The results were calculated using the dose-response sigmoidal curve (dependence of percentage free radical inhibition on AgNP concentration) in OriginPro8 software (OriginLab, Northampton, MA, USA) as described in Srećković et al. [74] and were given as IC_50_ values (µg/mL).

### 3.6. Antimicrobial Activity

Further analysis of the AgNPs’ biological potential continued with the testing of their antimicrobial activity. For that purpose, the microdilution method [77] was used to define the minimum inhibitory concentrations (MICs) of both types of AgNPs. In the tests of antibacterial activity, eleven bacterial species were used, namely *Micrococcus lysodeikticus* ATCC 4698, *Enterococcus faecalis* ATCC 29212, *Escherichia coli* ATCC 25922, *Klebsiella pneumoniae* ATCC 70063, *Pseudomonas aeruginosa* ATCC 10145, *Bacillus cereus* ATCC 10876, *Bacillus subtilis* ATCC 6633, *Salmonella enteritidis* ATCC 13076, *Salmonella typhimurium* ATCC 14028, *Staphylococcus epidermidis* ATCC 12228, and *Staphylococcus aureus* ATCC 25923. The antifungal potential of FUA-AgNPs and SVA-AgNPs was evaluated on eight fungal species, i.e., *Fusarium oxysporum* FSB 91, *Alternaria alternata* FSB 51, *Aspergillus brasiliensis* ATCC 16404, *Trichoderma lougibrachiatum* FSB 13, *Trichoderma harzianum* FSB 12, *Penicillium canescens* FSB 24, *Penicillium cyclopium* FSB 23, *Doratomyces stemonitis* FSB 41, and the yeast *Candida albicans* ATCC 10259. All microogranisms were from the Institute for Public Health in Kragujevac, Serbia and the Laboratory for Microbiology, Department of Biology and Ecology, Faculty of Science, University of Kragujevac. They were cultivated on nutrient agar (NA), potato glucose agar (PDA), and Sabouraud dextrose agar (SDA), respectively, and subcultured before the experiment.

For the microdilution assay, 96-well microtiter plates were used. Serial dilutions of samples and standards were made in Müller–Hinton broth (MHB) and Sabouraud dextrose broth (SDB) for bacterial and fungal cultures, respectively, following the previously reported procedure [22]. The concentrations of tested AgNPs were from 10 × 10^3^ to 39.1 µg/mL, while antibiotics (Ciprofloxacin and Clotrimazole) were applied at concentrations from 40 to 0.3125 µg/mL. The culture suspensions were made in 5% DMSO to an end concentration of 1.0 × 10^6^ CFU/mL for bacteria and *C. albicans* and 5 × 10^4^ CFU/mL for fungi [78,79]. After preparing double dilutions, the indicator solution of bacterial growth, resazurin (10 μL, 600 µg/ mL), was added into each of the wells of the plates used for bacterial cultures. The same volume of SBD was added in the plates used for antifungal analysis. In the end, 10 μL of microbial suspension was added and the plates were incubated at 37 °C for 24 h for bacteria and 28 °C for 72 h for fungi. Sterility and growth controls were made for each plate to monitor conditions that could affect the outcome of the trial. MICs were monitored visually for bacteria where the color of the indicator did not change, while a well without culture growth was detected for fungi.

### 3.7. MTT Cell Viability Assay

The normal human lung fibroblast cell line, MRC-5, the human breast cancer cell line, MDA-MB-231, the human placental choriocarcinoma JEG-3 cell line, the human colon cancer HCT-116 cell line human, and the chronic myelogenous leukemia K562 cell line were obtained from the American Tissue Culture Collection. These cells were propagated and maintained in DMEM supplemented with 10% FBS and with a mixture of antibiotics (100 IU/mL penicillin and 100 µg/mL streptomycin). The cells were grown in a 75 cm^2^ culture flask and supplied with 15 mL of DMEM at a confluence of 70 to 80%. The cells were seeded in a 96-well microplate (10,000 cells per well) and cultured in a humidified atmosphere with 5% CO_2_ at 37 °C. After 24 h of cell incubation, 100 μL of medium containing various doses of treatment (5 to 100 µg/mL) was added into each microplate well, and the cells were incubated for 24 and 72 h. Afterwards, the evaluation of cell viability was performed. Non-treated cells were used as control. The stock solution was prepared in a concentration of 10 × 10^3^ µg/mL, while the concentrations 5, 10, 20, 50, 100 µg/mL were used for assessment of cell viability during the experiment. The aforementioned concentrations were obtained from the stock solution by adding a compatible volume of DMEM. All concentrations were tested in triplicate, and cell viability was determined by MTT assay [80]. Briefly, the cells were plated at a density of 100,000 cells/mL (100 µL/well) in 96-well plates with DMEM. After a period of incubation (24 h), at a temperature of 37 °C and 5% CO_2_, the five different concentrations of two nanoparticles in concentration (5 to 100 µg/mL) were dissolved in DMEM and were added to each well (100 µL per well). The untreated cells (cultured only in a medium) served as a control. Both treated and control cells were incubated for 72 h, after which the cell viability was determined with MTT assay. After a period of incubation, 20 µL of MTT (concentration of 5 × 10^3^ µg/mL) was added to each well. MTT is a yellow tetrazolium salt that is reduced to purple formazan in the presence of mitochondrial dehydrogenase. During this reaction, which started approximately after three hours, the formed crystals were dissolved in 20 µL of DMSO. The color formed in the reaction was measured on an ELISA reader at a wavelength of 550 nm. The percentage of viable cells was calculated as the ratio between the absorbance at each dose of the treatment and the absorbance of the non-treated control multiplied by 100 to get a percentage. All data were evaluated using IBM-SPSS 23 software for Windows (SPSS Inc., Chicago, IL, USA). The data were presented as a mean ± standard error (S.E.M). The statistical significance was determined using a Paired Sample–T test. The level of statistical significance was set at * *p* < 0.05. We also calculated the half-maximal inhibitory concentration (IC_50_), defined as the concentration of tested nanoparticles that inhibited cell growth by 50% when compared to control. The IC_50_ values were calculated from the dose curves by the software CalcuSyn, Version 2.0. 

### 3.8. Catalytic Degradation of Congo Red

The catalytic properties of FUAgNP and SVAgNP were monitored in a reaction of Congo red (CR) reduction in the presence of NaBH_4_ [81]. According to the procedure, a 1 mL of FUAgNP or SVAgNP solution concentration of 100 µg/mL was added to a 5 mL of 10 µM CR solution and a 1.5 mL of 1 mM NaBH_4_ solution, so that the reaction rate of Congo red catalytic degradation at various time intervals was spectrophotometrically monitored (λmax 497 nm). The reaction took place in the dark, at room temperature, and at a neutral pH value. The reagent prepared immediately before the start of the reaction was used considering that NaBH_4_ is prone to degradation in neutral and acidic environments. The constant of reaction rate ‘*k*’ was calculated by the following Equation:

$$ln\left(\frac{A_{0}}{A_{t}}\right)=kt$$

where *A*_0_ is the absorbance at 0 min, *A_t_* is the absorbance at different times, ‘*k*’ is a constant of reaction, and *t* is the reaction time.

## 4. Conclusions

The study demonstrated that aqueous extracts of two plants with high phenolic content and antioxidant potential, *S. verticillata* and *F. ulmaria*, can be successfully used for rapid and eco-friendly synthesis of AgNPs with valuable biological activities. Nanoparticles obtained using these two plant species possessed similar characteristics and biological properties. Both AgNPs showed promising antimicrobial potential with pronounced antibacterial activity, as well as antioxidant activity with IC_50_ values below 80 µg/mL. FUAgNP and SVAgNP possessed full biocompatibility with the normal human lung fibroblast cell line MRC-5. However, AgNPs synthesized using *F. ulmaria* extract, may be distinguished as nanoparticles with lower particle diameter and better antimicrobial and antioxidant properties when compared with nanoparticles obtained using *S. verticillate* extract. Synthesized nanoparticles displayed a moderate decrease in cancer cell viability in concentrations up to 100 µg/mL, except for the colon cancer HCT-116 cell line where a 50% reduction in cell viability was noted at concentrations lower than 100 µg/mL. The cytotoxic potential of FUAgNP and SVAgNP against the HCT-116 cell line opens new possibilities for their research in cancer therapy. Their determined efficient catalytic properties in the degradation of azo dye Congo red highlight another ecological application of them. Both synthesized nanoparticles may find application as good antimicrobial and antioxidant substances for the development of new materials and formulations in the food industry, pharmacy, and medicine. In favor of their application for human use is also their high biocompatibility with the normal human fibroblast cell line (MRC-5) in concentrations that showed strong antioxidant activity and antibacterial effects on some pathogenic bacterial species.

## Figures and Tables

**Table 1:** Antimicrobial activity of synthesized nanoparticles.

Microorganisms		MIC (µg/mL)		
		FUAgNP	SVAgNP	Ciprofloxacin/ Clotrimazole ^a^
**Bacterial Strains**				
*S. aureus* *S. epidermidis* *B. cereus* *B. subtilis* *E. faecalis* *M. lysodeikticus* *E. coli* *S. typhimurium* *S. enteritidis* *K. pneumoniae* *P. aeruginosa*	G+	˂39.1	78.1	2.5
	G+	625.0	156.2	2.5
	G+	˂39.1	˂39.1	20
	G+	˂39.1	78.1	10
	G+	˂39.1	˂39.1	˂0.3125
	G+	625.0	156.2	˂0.3125
	G−	625.0	2500	˂0.3125
	G−	78.1	78.1	5
	G−	˂39.1	˂39.1	˂0.3125
	G−	˂39.1	˂39.1	˂0.3125
	G−	˂39.1	78.1	˂0.3125
**Fungal Strains**				
*C. albicans*		312.5	312.5	10
*A. brasiliensis*		˃10 × 10^3^	˃10 × 10^3^	1.25
*P. canescens*		˂78.1	˂78.1	2.5
*P. cyclopium*		˂78.1	˂78.1	˂0.0391
*T. lougibrachiatum*		˂78.1	312.5	20
*T. harzianum*		1250	1250	40
*F. oxysporum*		˃10 × 10^3^	˃10 × 10^3^	˂0.0391
*D. stemonitis*		5 × 10^3^	5 × 10^3^	0.625
*A. alternata*		˃10 × 10^3^	˃10 × 10^3^	˂0.0391

^a^ Ciprofloxacin was used as reference antibiotic for bacteria, while clotrimazole was used for fungi.

## Data Availability

Data sharing not applicable.

## Sample Availability

Samples of the synthesized nanoparticles are available from the authors.
